# Association of central adiposity with psoriasis, psoriatic arthritis and rheumatoid arthritis: a cross-sectional study of the UK Biobank

**DOI:** 10.1093/rheumatology/kez192

**Published:** 2019-05-25

**Authors:** Lyn D Ferguson, Rosemary Brown, Carlos Celis-Morales, Paul Welsh, Donald M Lyall, Jill P Pell, Iain B McInnes, Stefan Siebert, Naveed Sattar

**Affiliations:** 1 Institute of Cardiovascular and Medical Sciences, Immunity and Inflammation, University of Glasgow, Glasgow, UK; 2 Institute of Health & Wellbeing, Immunity and Inflammation, University of Glasgow, Glasgow, UK; 3 Institute of Infection, Immunity and Inflammation, University of Glasgow, Glasgow, UK

**Keywords:** central adiposity, waist circumference, psoriasis, psoriatic arthritis, rheumatoid arthritis

## Abstract

**Objectives:**

To determine the independent association of central adiposity, assessed by waist circumference, with odds of psoriasis, PsA and RA prevalence after controlling for general adiposity (BMI).

**Methods:**

A cross-sectional study of UK Biobank participants aged 40–70 years was performed. Logistic regression was used to calculate the odds of psoriasis, PsA and RA occurrence compared with controls without these conditions by waist circumference, adjusting for covariates: age, sex, smoking status, socioeconomic deprivation and self-reported physical activity (Model 1), followed additionally by BMI (Model 2).

**Results:**

A total of 502 417 participants were included; 5074 with psoriasis (1.02%), 905 with PsA (0.18%), 5532 with RA (1.11%) and 490 906 controls without these conditions. Adjusted odds ratios (ORs) (Model 1) for psoriasis, PsA and RA, per s.d. (13.5 cm) higher waist circumference were 1.20 (95% CI 1.16, 1.23), 1.30 (95% CI 1.21, 1.39) and 1.21 (95% CI 1.17, 1.24), respectively (all *P* < 0.001). These ORs remained significant after further adjustment for BMI (Model 2) in psoriasis [OR 1.19 (95% CI 1.12, 1.27), *P* < 0.001] and RA [OR 1.19 (95% CI 1.12, 1.26), *P* < 0.001], but not in PsA [OR 1.11 (95% CI 0.95, 1.29), *P* = 0.127].

**Conclusion:**

Central adiposity as measured by waist circumference is associated with greater odds of psoriasis and RA prevalence after adjustment for confounders and for BMI. Our findings add support for central adiposity as a long-term clinically relevant component of these conditions.

**Rheumatology key messages**

- Central adiposity is associated with greater odds of psoriasis and RA independently of BMI.
- These data add further insight into the relationship of body fat distribution in autoimmune disease.
- More trials assessing clinical benefits of intentional weight loss in autoimmune conditions would be beneficial.

## Introduction

Observational studies have shown a positive association between increased BMI and psoriasis and PsA incidence [1, 2]. Recent Mendelian randomization showed that higher BMI increased the odds of psoriasis [odds ratio (OR) 1.09 per 1 kg/m^2^], with limited evidence for causality in the opposite direction [3]. There appears to be a direct link between adiposity and inflammation, with greater adiposity allele scores associated with higher CRP levels [4]. The traditional image of rheumatoid cachexia leading to lower BMIs in RA [5] has now been somewhat expanded, with recent studies reporting an association between higher BMI and greater RA development [6, 7], particularly in ACPA-negative RA [8]. Increased fat mass has been correlated with disease activity with postulated links through pro-inflammatory adipokine production [9]. However, other studies have shown no association between BMI and RA [10, 11], which may reflect measuring BMI at different stages of the disease.

While BMI is often used to assess general adiposity, evidence supports central adiposity measures, namely waist circumference, as potentially stronger predictors of future cardiometabolic risk, including diabetes risk in women [12] and stroke risk [13]. Waist circumference is more linearly associated with incident cardiovascular disease risk than BMI, and far less subject to reverse causality [14].

No studies to date have simultaneously examined whether individuals with psoriasis, PsA and RA have greater central adiposity relative to those without these conditions. We determined the independent association of central adiposity assessed by waist circumference with odds of psoriasis, PsA and RA occurrence after controlling for general adiposity (BMI) and other confounding factors in a large population cohort, the UK Biobank, comprising ∼500 000 subjects.

## Methods

### Study design and participants

The UK Biobank is a large, population-based cohort study set up to study lifestyle, environmental and genetic determinants of adulthood diseases. Between April 2007 and December 2010, UK Biobank recruited 502 682 participants aged 40–70 years from the general population. Participants attended 1 of 22 assessment centres across England, Wales and Scotland, where they completed touch-screen questionnaires, had physical measurements taken and provided biological samples [15]. The present study used baseline data to investigate cross-sectional association between waist circumference and psoriasis, PsA and RA occurrence compared with those not reporting these outcomes. The UK Biobank study was approved by the North West Multicentre Ethics Research Committee; participants provided written informed consent for data collection and analysis. This study is part of UK Biobank project 3966 (NHS National Research Ethics Service Ref 11/NW/0382).

### Outcomes, exposures and covariates

Outcomes were self-reported diagnosis of psoriasis, PsA or RA. Participants were classified as psoriasis if they reported psoriasis only (and not PsA or RA), PsA if they reported PsA or PsA and psoriasis, and RA if they reported RA only (and not PsA); those who reported both RA and PsA were omitted. The exposure variable was waist circumference. Covariates included: age, sex, socioeconomic deprivation index, smoking status, physical activity and BMI.

Anthropometric measurements were obtained by trained personnel following standard operating procedures and using calibrated equipment. Weight was measured, without shoes and outdoor clothing, using the Tanita BC 418 body composition analyser. Height was measured, without shoes, using the wall-mounted SECA 240 height measure. BMI was calculated from weight (in kilograms) divided by square of height (in meters). Waist circumference was measured midway between lowest rib margin and iliac crest, in a horizontal plane, using a non-elastic SECA 200 tape measure. Further details can be found in the UK Biobank protocol [16].

Socioeconomic status was measured using the Townsend deprivation score, an area of residence-based index of material deprivation derived from census information on housing, employment, social class and car availability [17]. Smoking status was categorized into never, former and current smoker. Physical activity was based on self-report, using the International Physical Activity Questionnaire (IPAQ) short form, and total physical activity was computed as the sum of walking, moderate and vigorous activity, measured as metabolic equivalents (MET-min/week) [18].

### Statistical analysis

All analyses were performed using statistical software STATA 14 (StataCorp LP, Texas, USA). Continuous data were presented as mean and s.d., categorical data as number (*n*) and percentage (%). Continuous variables were checked for normality by visual inspection of histograms. Logistic regression was used to calculate odds of psoriasis, PsA and RA compared with controls per s.d. (13.5 cm) higher waist circumference and by waist circumference quintiles, adjusting for confounders: age, sex, smoking status, socioeconomic deprivation and physical activity (Model 1), followed additionally with adjustment for BMI (Model 2). Waist circumference quintiles for men were: lowest quintile ⩽88 cm, lower-middle 88.1–93 cm, middle 93.1–99 cm, middle-higher 99.1–105 cm, highest quintile >105 cm; for women: lowest quintile ⩽74 cm, lower-middle 74.1–80 cm, middle 80.1–86 cm, middle-higher 86.1–95 cm, highest quintile >95 cm.

Due to the differential distribution of adipose tissue between men and women, a sex-stratified analysis was conducted, with a formal interaction between waist circumference and sex for psoriasis, PsA and RA tested by fitting an interaction term to the model e.g. ‘waistT5#sex’. A separate sensitivity analysis was also performed by excluding individuals with significant comorbidities (*n* = 130 991) including: chronic obstructive pulmonary disease, asthma, heart disease, chronic liver disease, depression, alcohol misuse, substance misuse, eating disorder, schizophrenia, Parkinson’s disease, dementia and cancer.

## Results

A total of 502 417 participants were included: 5074 with psoriasis (1.02%), 905 with PsA (0.18%), 5532 with RA (1.11%), and 490 906 controls without self-reported psoriasis, PsA or RA. Baseline characteristics are outlined in Table 1. RA participants were older and predominantly female compared with controls. Among RA individuals, 25.2% were in the most socioeconomically deprived quintile, compared with 19.9% of controls. Those with psoriasis had the greatest percentage of current smokers (16.3%). Mean BMI and waist circumference were higher in men and women in all three disease groups compared with controls. Self-reported physical activity levels were lower in all three disease groups in men compared with controls and in women with PsA and RA compared with controls (Table 1).

**Table 1:** Baseline characteristics of psoriasis, PsA and RA participants compared with controls

	Controls (*n* = 490 906)	Psoriasis (*n* = 5074)	PsA (*n* = 905)	RA (*n* = 5532)
Age, mean (s.d.), years	56.5 (8.1)	56.4 (8.1)	56.2 (7.4)	59.2 (7.1)
Sex, *n* (%)				
Female	266 673 (54)	2363 (47)	464 (51)	3872 (70)
Male	224 304 (46)	2711 (53)	441 (49)	1660 (30)
Smoking status, *n* (%)				
Current smoker	51 318 (10.5)	825 (16.3)	94 (10.4)	696 (12.6)
Ex-smoker	239 828 (49.0)	2688 (53.0)	453 (50.1)	2781 (50.4)
Non-smoker	198 582 (40.6)	1556 (30.7)	357 (39.5)	2037 (36.9)
Deprivation quintile, *n* (%)				
1 (least)	98 555 (20.1)	933 (18.4)	178 (19.7)	975 (17.7)
2	98 002 (20.0)	931 (18.4)	192 (21.3)	959 (17.4)
3	98 131 (20.0)	1008 (19.9)	165 (18.3)	1060 (19.2)
4	98 007 (20.0)	1043 (20.6)	174 (19.3)	1138 (20.6)
5 (most)	97 601 (19.9)	1158 (22.8)	194 (21.5)	1391 (25.2)
BMI, mean (s.d.), kg/m^2^				
Men	27.8 (4.2)	28.5 (4.6)	28.6 (4.4)	28.5 (4.7)
Women	27.1 (5.2)	27.9 (5.5)	28.9 (6.1)	28.2 (5.8)
Waist circumference, mean (s.d.), cm				
Men	96.9 (11.3)	99.0 (12.0)	98.7 (11.0)	99.7 (12.1)
Women	84.7 (12.5)	86.9 (13.2)	89.2 (14.2)	87.8 (13.6)
Physical activity, mean (s.d.), total METmin/week^a^				
Men	2763 (3331)	2563 (3204)	2156 (2777)	2487 (3472)
Women	2321 (2801)	2230 (2794)	1977 (2682)	1935 (2748)

a Metabolic equivalent task (MET) min/week.

**Fig. 1:**
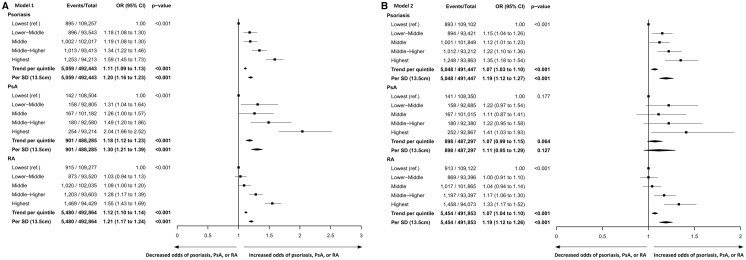
Odds of psoriasis, PsA, and RA with increasing waist circumference (**A**) Odds of psoriasis, PsA and RA adjusted for: age, sex, socioeconomic deprivation quintile, smoking status and physical activity (Model 1). (**B**) Odds of psoriasis, PsA and RA fully adjusted for the above covariates plus BMI (Model 2). Waist circumference quintiles for men: lowest quintile ≤88 cm, lower-middle 88.1–93 cm, middle 93.1–99 cm, middle-higher 99.1–105 cm, highest quintile >105 cm. Waist circumference quintiles for women: lowest quintile ≤74 cm, lower-middle 74.1–80 cm, middle 80.1–86 cm, middle-higher 86.1–95 cm, highest quintile >95 cm.

The adjusted ORs (Model 1) for psoriasis, PsA and RA, per s.d. (13.5 cm) higher waist circumference were 1.20 (95% CI 1.16, 1.23), 1.30 (95% CI 1.21, 1.39) and 1.21 (95% CI 1.17, 1.24), respectively (all *P* < 0.001) (Fig. 1A). These odds remained significant after further adjustment for BMI (Model 2) in psoriasis [OR 1.19 (95% CI 1.12, 1.27), *P* < 0.001] and RA [OR 1.19 (95% CI 1.12, 1.26), *P* < 0.001], but not in PsA [OR 1.11 (95% CI 0.95, 1.29), *P* = 0.127] (Fig. 1B).

Analysis by waist circumference quintiles revealed adjusted ORs (Model 1) for psoriasis, PsA and RA, in the highest compared with lowest waist circumference quintiles of: 1.59 (95% CI 1.45, 1.73), 2.04 (95% CI 1.66, 2.52) and 1.55 (95% CI 1.43, 1.69), respectively (Fig. 1A). Full adjustment including BMI (Model 2) attenuated these ORs to 1.35 (95% CI 1.18, 1.54), 1.41 (95% CI 1.03, 1.93) and 1.33 (95% CI 1.17, 1.52), respectively (Fig. 1B).

Sensitivity analysis excluding significant comorbidities showed broadly similar results (supplementary Table S1, available at *Rheumatology* online). There was no statistical interaction between sex and waist circumference in psoriasis and RA; however, there was evidence of an interaction in PsA. Due to known differences in body fat distribution between men and women, stratified analyses by sex are presented in supplementary Figs S1 and S2, available at *Rheumatology* online. The odds of psoriasis per s.d. higher waist circumference were similar in men and women. Associations with RA were somewhat stronger for men than for women after BMI adjustment. Odds of PsA per s.d. higher waist circumference appeared higher in women than men, although this was not statistically significant after BMI adjustment. These results should be interpreted with caution due to the smaller sample size and wider CIs in these smaller subgroups.

## Discussion

To our knowledge, this is the first study to simultaneously examine the role of central adiposity in psoriasis, PsA and RA occurrence compared with controls in a large population-based dataset. Higher waist circumferences were associated with greater odds of psoriasis, PsA and RA, with greatest relative odds for PsA occurrence. The association remained significant in psoriasis and RA after adjustment for general adiposity (BMI); while the point estimate remained elevated in PsA, this was no longer statistically significant after BMI adjustment.

The Nord-Trøndelag Health (HUNT) study reported that psoriasis risk almost doubled in the highest compared with lowest waist circumference quartile [1]. However, they did not adjust for BMI, were limited to 369 incident psoriasis cases, and had no information on PsA or RA. The Nurses’ Health Study also showed positive association between waist circumference and psoriasis risk [19]. In contrast to our study, after BMI adjustment this became non-significant. Anthropometric measurements were, however, self-reported and based on 809 psoriasis cases, whereas in UK Biobank all measurements were made by trained personnel in >5000 individuals with psoriasis.

While some studies have suggested that obesity was associated with over triple the odds of developing RA [20], others have shown no increased risk [10]. Meta-analysis of 11 studies revealed that compared with those with a BMI <30 kg/m^2^, those who were obese had a significantly increased risk of RA (relative risk 1.25, 95% CI 1.07, 1.45); however, there was significant heterogeneity between studies [21]. By using waist circumference and adjusting for BMI, we have shown an association between this central adiposity marker and higher odds of RA occurrence. While this study is cross-sectional and cannot demonstrate causality, it may be that waist circumference rises in those with prevalent disease due to the disease process itself, or those with greater central adiposity are more prone to developing autoimmune disease.

Obesity is associated with increased PsA risk [2]; our results confirm this, with the strongest association between higher waist circumference and PsA out of the three conditions studied. After BMI adjustment, this association was no longer statistically significant. However, the PsA group was considerably smaller than psoriasis and RA groups, reducing the ability to detect significant associations in adjusted analyses.

Stratified analyses by sex showed similar associations in psoriasis in men and women, with the suggestion of a stronger association of waist circumference with PsA in women. However, this latter result should be interpreted with caution due to smaller sample size and wider CIs in this group. Associations with RA appeared stronger in men after BMI adjustment. Such sex differences were also noted by Ljung *et al.* who showed an >3-fold increased risk of developing RA in men with abdominal obesity (waist circumference >102 cm) [22].

Study strengths include robust measurement of waist circumference and BMI and the large sample size of the UK Biobank, one of the largest studies to simultaneously examine central adiposity in psoriasis, PsA and RA compared with controls. Sensitivity analysis excluding significant comorbidities demonstrated the robustness of results. Limitations include the cross-sectional study design. The UK Biobank is not entirely representative of the whole UK population, with evidence of a healthy volunteer selection bias [23]. This may partly explain the lower prevalence of psoriasis in this study (1.02%) compared with that reported in other studies such as the UK Clinical Practice Research Datalink (CPRD) (reported psoriasis prevalence 2.8%) [24]. Potential misclassification bias due to the self-reported nature of diagnoses may potentially have led to over-reporting of RA in the UK Biobank (prevalence 1.11%), compared with that reported by Symmons *et al.* [25] (estimated RA prevalence 0.81% in UK). However, the valid assessment of exposure–disease relationships in UK Biobank is still widely generalizable [23].

In conclusion, higher waist circumference was associated with higher odds of psoriasis, PsA and RA prevalence compared with controls. Importantly, this remained significant in psoriasis and RA after BMI adjustment, highlighting the potential importance of central adiposity in these autoimmune conditions. This is the largest study to examine such associations and, given established links of central adiposity to adverse cardiometabolic outcomes [12, 14], suggests better understanding of the link between altered body composition and risk for autoimmune conditions is needed. The potential for autoimmune processes to lead to altered body composition should also be borne in mind. Either way, our findings add stronger support for central adiposity as a relevant player in the long-term complications of these conditions. They also suggest a need to consider more trials of weight loss interventions in autoimmune conditions, especially as the evidence base for lifestyle-induced weight loss has substantially improved in recent years.

## Supplementary Material

kez192_Supplementary_Data
