# Supplementary material for: Association of central adiposity with psoriasis, psoriatic arthritis and rheumatoid arthritis: a cross-sectional study of the UK Biobank
Source: Rheumatology (Oxford). 2019 May 25;58(12):2137–42. doi: 10.1093/rheumatology/kez192 (PMC6880847; doi:10.1093/rheumatology/kez192)
Supplement: kez192_Supplementary_Data [file kez192_supplementary_data.zip › kez192-Suppl_data/Supplementary_Data.docx]

**SUPPLEMENTARY MATERIAL**

Supplementary Table S1. Odds of Psoriasis, PsA, & RA with increasing waist circumference in participants without significant comorbidities^a^.

|  | Psoriasis (n=3,678) | | PsA (n=682) | | RA (n=3,695) | |
| --- | --- | --- | --- | --- | --- | --- |
|  | **Adjusted OR^b^**  **(95% CI)** | **Fully adjusted OR^c^**  **(95% CI)** | **Adjusted OR^b^**  **(95% CI)** | **Fully adjusted OR^c^ (95% CI)** | **Adjusted OR^b^ (95% CI)** | **Fully adjusted OR^c^ (95% CI)** |
| Per SD (13.5cm) | 1.18 (1.14-1.23) (<0.001) | 1.22 (1.12-1.31) (<0.001) | 1.27 (1.17-1.38) (<0.001) | 1.11 (0.93-1.33) (0.195) | 1.20 (1.15-1.24) (<0.001) | 1.15 (1.06-1.24) (<0.001) |
| Quintiles^d^ |  |  |  |  |  |  |
| First | 1.00 (Ref) (<0.001) | 1.00 (Ref)  (0.003) | 1.00 (Ref)  (<0.001) | 1.00 (Ref)  (0.414) | 1.00 (Ref) (<0.001) | 1.00 (Ref)  (0.004) |
| Second | 1.20 (1.08-1.33) | 1.17 (1.05-1.31) | 1.32 (1.02-1.70) | 1.23 (0.95-1.60) | 1.01 (0.90-1.12) | 0.96 (0.86-1.08) |
| Third | 1.19 (1.08-1.33) | 1.15 (1.02-1.28) | 1.34 (1.05-1.72) | 1.19 (0.91-1.55) | 1.07 (0.96-1.19) | 1.00 (0.89-1.12) |
| Fourth | 1.33 (1.19-1.47) | 1.24 (1.09-1.41) | 1.36 (1.06-1.76) | 1.12 (0.83-1.52) | 1.25 (1.13-1.39) | 1.13 (0.99-1.28) |
| Fifth | 1.51 (1.36-1.68) | 1.35 (1.14-1.59) | 1.89 (1.49-2.41) | 1.32 (0.91-1.91) | 1.49 (1.35-1.65) | 1.23 (1.05-1.45) |
| Trend | 1.10 (1.07-1.12) (<0.001) | 1.07 (1.03-1.11) (<0.001) | 1.14 (1.08-1.21) (<0.001) | 1.04 (0.96-1.13) (0.285) | 1.11 (1.08-1.14) (<0.001) | 1.05 (1.02-1.09) (0.003) |

^a^ n=130,991 individuals were excluded with the following co-morbidities: chronic obstructive pulmonary disease COPD, asthma, heart disease, chronic liver disease, depression, alcohol misuse, substance misuse, eating disorder, schizophrenia, Parkinson’s disease, dementia, and cancer diagnosis. ^b^Model 1: adjusted for age, sex, smoking status, socioeconomic deprivation quintile, and physical activity. ^c^Model 2: adjusted for covariates in model 1 plus Body Mass Index (BMI). ^d^Waist circumference quintile ranges for men: lowest quintile ≤88cm, lower-middle 88.1-93cm, middle 93.1-99cm, middle-higher 99.1-105cm, highest quintile >105cm; for women: lowest quintile ≤74cm, lower-middle 74.1-80cm, middle 80.1-86cm, middle-higher 86.1-95cm, highest quintile >95cm. OR: odds ratio; 95% CI: confidence interval.

**Supplementary figure legends**

**Supplementary Figure S1 Odds of psoriasis, PsA, and RA with increasing waist circumference in men**

A: Odds of psoriasis, PsA, and RA adjusted for: age, sex, socioeconomic deprivation quintile, smoking status, and physical activity (model 1). B: Odds of psoriasis, PsA, and RA fully adjusted for above covariates plus body mass index (BMI) (model 2). Waist circumference quintiles: lowest quintile ≤88cm, lower-middle 88.1-93cm, middle 93.1-99cm, middle-higher 99.1-105cm, highest quintile >105cm.

**Supplementary Figure S2 Odds of psoriasis, PsA, and RA with increasing waist circumference in women**

A: Odds of psoriasis, PsA, and RA adjusted for: age, sex, socioeconomic deprivation quintile, smoking status, and physical activity (model 1). B: Odds of psoriasis, PsA, and RA fully adjusted for above covariates plus body mass index (BMI) (model 2). Waist circumference quintiles: lowest quintile ≤74cm, lower-middle 74.1-80cm, middle 80.1-86cm, middle-higher 86.1-95cm, highest quintile >95cm.
