# Supplementary figures and images for: Association of central adiposity with psoriasis, psoriatic arthritis and rheumatoid arthritis: a cross-sectional study of the UK Biobank
Source: Rheumatology (Oxford). 2019 May 25;58(12):2137–42. doi: 10.1093/rheumatology/kez192 (PMC6880847; doi:10.1093/rheumatology/kez192)

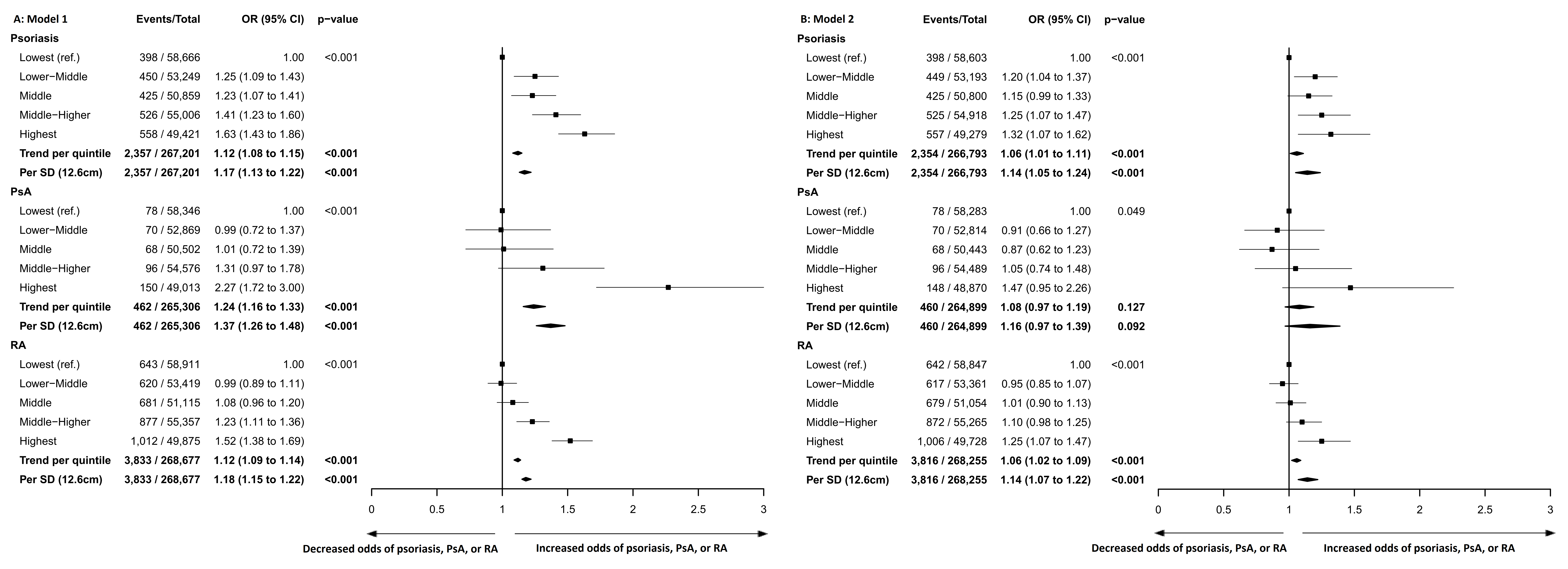

Supplement: kez192_Supplementary_Data [file kez192_supplementary_data.zip › kez192-Suppl_data/Supplementary_Figure2.tif]

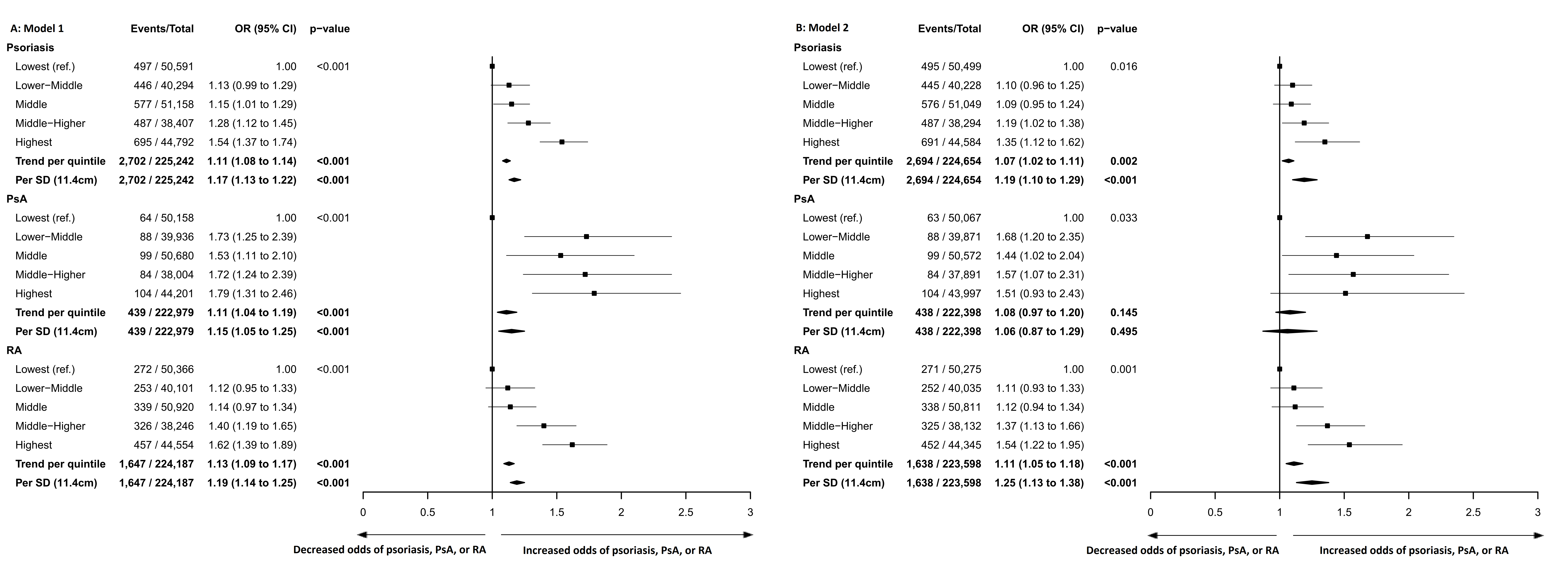

Supplement: kez192_Supplementary_Data [file kez192_supplementary_data.zip › kez192-Suppl_data/Supplementary_Figure1.tif]
